# DCSF-Net: a dual-branch cross-scale spatiotemporal fusion network for fine-scale mangrove mapping from RGB and Sentinel-2 imagery

**DOI:** 10.3389/fpls.2026.1931676

**Published:** 2026-08-24

**Authors:** Shiyu Zhuang, Lili Wen, Yushen Wang, Zhihan Wang, Yiduo He, Lei Chen, Kailong Cui

**Affiliations:** 1 College of Marine Science and Environment, Dalian Ocean University, Dalian, China; 2 Guangxi Key Laboratory of Marine Environmental Science, Guangxi Academy of Marine Sciences, Nanning, China; 3 The State Key Laboratory of Information Engineering in Surveying, Mapping and Remote Sensing, Wuhan University, Wuhan, China

**Keywords:** DCSF-Net, mangrove mapping, RGB-Sentinel-2 fusion, semantic segmentation, spectral-phenological features

## Abstract

Accurate fine-scale mangrove mapping is essential for coastal resource monitoring, ecological restoration, and shoreline management, yet fragmented boundaries, heterogeneous intertidal backgrounds, and the limited spectral dimensionality of high-resolution RGB imagery continue to constrain class separability. We developed DCSF-Net, a dual-branch cross-scale spatiotemporal fusion network that integrates 0.3 m RGB imagery with a 26-channel Sentinel-2 spectral-phenological feature tensor derived from 2024 dry-season (T1, October-December) and wet-season (T2, April-September) median composites and their differences. The RGB branch uses local boundary attention (LBA) and a directional topology module (DTM) to retain local contrast and elongated morphology, while the Deformable Cross-Scale Attention Bridge (D-CSAB) fuses the high-resolution spatial feature tensor output by the RGB branch with the upsampled Sentinel-2 representation through RGB-guided deformable convolution, cross-attention, and zero-initialized residual injection. On the test set, DCSF-Net achieved an F1-score of 95.68% and an intersection over union (IoU) of 91.72%, outperforming the evaluated RGB-only baselines. In matched-input ablations, replacing D-CSAB with direct fusion reduced IoU by 3.20 percentage points, removing zero-initialized residual injection reduced IoU by 1.86 points, and jointly removing LBA and DTM reduced IoU by 5.36 points. These results demonstrate the value of combining fine spatial detail with complementary spectral-seasonal context for mangrove mapping within the present study area and experimental design.

## 1 Introduction

Mangroves are typical woody wetland communities distributed in tropical and subtropical coastal intertidal zones. They play important roles in coastal protection, carbon storage, biodiversity maintenance, and land-sea material cycling (Giri et al., 2011*;* Donato et al., 2011*;* Alongi, 2020). At the same time, coastal development, land reclamation, aquaculture expansion, and climate change continue to alter mangrove habitats, resulting in degradation, fragmentation, and dynamic boundary changes (Friess et al., 2019*;* Bryan-Brown et al., 2020*;* Goldberg et al., 2020*;* Richards and Friess, 2016). Therefore, fine-scale, reliable, and repeatedly updatable information on mangrove spatial distribution is an important basis for resource monitoring, ecological restoration assessment, and coastal zone management.

Traditional field surveys can provide reliable sample information, but limitations in survey extent, tidal windows, and labor costs make them insufficient for large-area and high-frequency monitoring. Satellite remote sensing has become an important technique for mangrove mapping and dynamic monitoring. Global mangrove products and remote-sensing reviews have shown that sensor resolution, imaging season, and classification strategy directly affect the identification of mangrove boundaries and small patches (Bunting et al., 2018*;* Bunting et al., 2022*;* Kuenzer et al., 2011*;* Heumann, 2011). Early studies mainly used object-based classification, vegetation-index thresholds, or traditional machine-learning methods. However, these methods depend on manually designed features and are easily affected by noise and background heterogeneity in areas where tidal flats, water bodies, shadows, and nearshore vegetation are intermingled (Maurya et al., 2021).

With the development of deep learning, semantic segmentation models can automatically learn hierarchical spatial semantic features from imagery and have been widely applied to remote-sensing land-cover classification and ecological-environment monitoring tasks (Zhu et al., 2017*;* Ma et al., 2019*;* Yuan et al., 2020a). The development of high-spatial-resolution imagery has substantially improved the representation of mangrove patch boundaries, canopy texture, and narrow belt-like structures (Zhang et al., 2025*;* Hong et al., 2024*;* Wang et al., 2024a). However, for Google Earth, unmanned aerial vehicle, or commercial high-resolution RGB imagery, the advantage lies in depicting sub-meter spatial details, whereas the limitation is that such imagery usually contains only visible bands. It is therefore difficult to fully distinguish mangroves from spectrally similar objects, such as other coastal vegetation, Spartina alterniflora, bare flats, shadows, and turbid water bodies (Li et al., 2020*;* Chen and Shi, 2023).

Fine-scale mangrove mapping is therefore a coupled spatial-spectral-scale problem. At the target mapping scale, 0.3 m RGB imagery resolves canopy texture, small fragments, narrow belts, tidal-channel edges, and shoreline context, but its three visible bands provide limited discrimination between mangroves and spectrally similar coastal backgrounds. Sentinel-2 contributes red-edge, near-infrared, shortwave-infrared, water-related, and seasonal information, but its native 10–20 m pixels mix multiple surface types. Effective fusion must therefore preserve the RGB grid as the source of output geometry while using Sentinel-2 observations as complementary semantic context.

Existing approaches address only part of this problem. RGB-only segmentation networks can model fine spatial context but remain vulnerable to coastal backgrounds with similar visible appearance. Sentinel-based mapping improves spectral discrimination but is constrained by mixed pixels in fragmented stands and narrow belts. Simple channel concatenation assumes that nominally coregistered features are already semantically aligned, whereas late decision fusion cannot adjust mismatches within the learned representation. A feature-level strategy that combines boundary-aware RGB encoding with content-adaptive cross-scale fusion is therefore needed.

To address this need, we developed DCSF-Net as a task-oriented integration of complementary spatial and spectral-seasonal information. The study makes three main contributions. First, paired inputs combine a 3-channel 0.3 m RGB patch with a 26-channel two-season Sentinel-2 spectral-phenological tensor over the same 76.8 m ground footprint. Second, the RGB encoder integrates LBA and DTM in its shallow and middle stages to enhance local contrast and directional morphology in fragmented and elongated mangrove patches. Third, after the RGB encoder-decoder generates the high-resolution spatial feature tensor, D-CSAB couples it with the upsampled Sentinel-2 feature through RGB-guided offset prediction, deformable convolution, RGB-query/Sentinel-key-value cross-attention, and zero-initialized residual injection. The contribution lies in this problem-specific organization and coupling of established operators within a dual-source mangrove segmentation framework.

The complete system was compared with nine RGB-only segmentation networks, while matched-input ablations examined D-CSAB relative to direct fusion, the role of zero-initialized residual injection, and the joint contribution of LBA and DTM. Genuine held-out predictions were further analyzed across continuous stands, fragmented patches, land-water interfaces, and narrow belts.

## 2 Study area and data

### 2.1 Study area

The Guangdong-Hong Kong-Macao Greater Bay Area is located along the Pearl River Estuary and its surrounding coastal areas in southern China. It faces the South China Sea and is bordered by inland Guangdong to the north. It is one of the regions in China with a high level of urbanization and intensive coastal development. The region lies in the southern subtropical monsoon climate zone and is strongly affected by estuarine runoff, tidal processes, and land-sea interactions. Its indented coastline and well-developed river-network and bay systems provide suitable hydrological and geomorphological conditions for the formation and growth of mangrove wetlands. In terms of spatial distribution, mangroves in the Greater Bay Area are mainly concentrated in Shenzhen Bay, Nansha in Guangzhou, Qi’ao Island in Zhuhai, Huangmao Sea, and the adjacent estuarine and bay intertidal zones, showing an overall belt-like distribution along coastlines and estuarine wetlands (Bunting et al., 2022).

**Figure 1:**
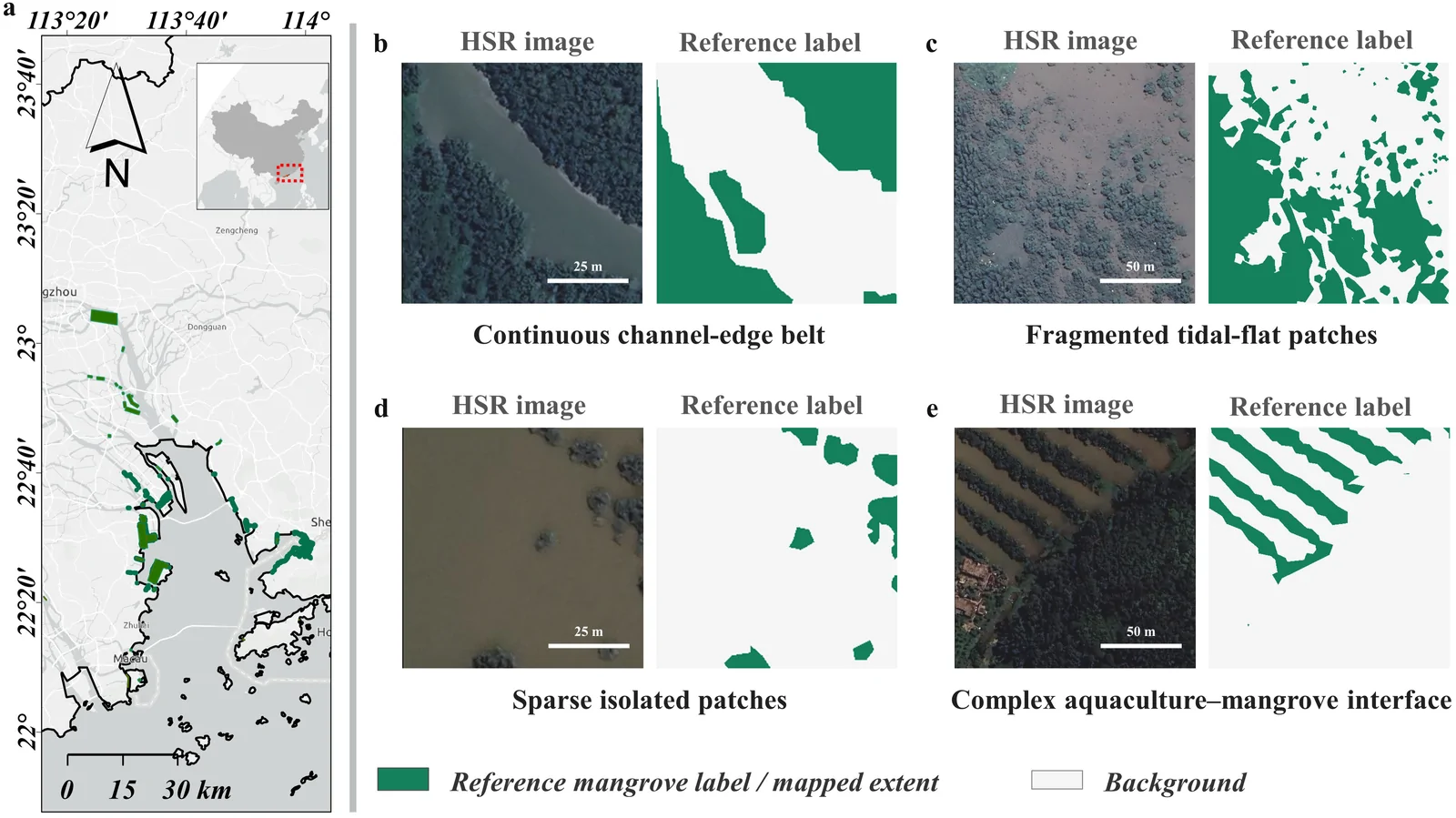
Location of the study area. **(a)** Geographic distribution of the sampled mangrove areas in the Guangdong-Hong Kong-Macao Greater Bay Area. **(b–e)** High-resolution RGB imagery and corresponding mangrove reference labels for a continuous channel-edge belt, fragmented tidal-flat patches, sparse isolated patches, and a complex aquaculture-mangrove interface, respectively.

To ensure the representativeness of the sample data, multiple typical mangrove distribution areas within the Guangdong-Hong Kong-Macao Greater Bay Area were selected as study sample areas (**Figure 1**). These sample areas cover different intertidal environments and human-disturbance backgrounds, and can reflect differences in mangrove spatial morphology, patch fragmentation, and surrounding land-cover composition across the Greater Bay Area. They provide the basis for subsequent dual-source data construction and model validation.

### 2.2 Data and preprocessing

This study integrated 0.3 m high-resolution RGB imagery with two 2024 Sentinel-2 seasonal composites to construct paired inputs for binary mangrove segmentation. RGB imagery provides fine spatial morphology, whereas Sentinel-2 provides coarser multispectral and seasonal context. Ten spectral bands, three indices, and their interseasonal differences were organized into a 26-channel Sentinel-2 spectral-phenological feature tensor.

The two sources were assigned complementary roles. The RGB branch defines the spatial lattice for reference labels and final predictions, while Sentinel-2 supplies contextual evidence related to vegetation condition, moisture, water adjacency, and seasonal spectral variation. Upsampling the Sentinel-2 tensor enables feature fusion at the network interface but does not increase the native information content of the 10–20 m observations.

### 2.3 Google high-resolution RGB imagery

Google Earth high-resolution RGB imagery provided the spatial-detail input. The imagery has an approximate spatial resolution of 0.3 m and three visible channels (red, green, and blue). Images were selected to minimize cloud shadow and severe blur while retaining clearly interpretable canopy texture and shoreline context. The RGB imagery used for sample construction was acquired in October 2025.

All spatial products used for paired-sample construction were represented in WGS 84/UTM zone 49N (EPSG:32649). A non-overlapping fishnet was placed on the 0.3 m RGB grid, and each cell contained 256 × 256 RGB pixels, corresponding to 76.8 × 76.8 m on the ground. The stride equalled the patch width (256 pixels or 76.8 m), so the stored RGB and reference-label windows had 0% overlap. For each fishnet cell, the aligned Sentinel-2 feature stack was cropped using the identical projected ground extent and then represented as a 26 × 256 × 256 tensor. The paired model inputs were therefore a 3 × 256 × 256 RGB tensor and a 26 × 256 × 256 Sentinel-2 tensor describing the same nominal footprint.

This procedure establishes correspondence at three levels: a common projected coordinate reference system, an identical ground-footprint envelope for synchronous cropping, and an identical tensor size at the network interface. It does not imply that each 0.3 m cell contains an independent Sentinel-2 observation. Multiple RGB cells share the support of a native Sentinel-2 pixel, particularly for the 20 m bands. Because the GEE script did not call an explicit resampling function, Earth Engine’s default nearest-neighbor resampling was used when the native 20 m bands were exported to the 10 m grid. The resulting Sentinel-2 stack was then aligned with the RGB grid for paired-sample construction.

### 2.4 Sentinel-2 dual-temporal multispectral composites

Sentinel-2 multispectral imagery is provided by the European Space Agency and is characterized by wide coverage, a short revisit cycle, and rich band information. This study selected Sentinel-2 images covering the same areas as the high-resolution RGB imagery. Blue, green, red, near-infrared, red-edge, and shortwave-infrared bands were used to construct the multispectral input, and vegetation indices and water-related indices were further calculated to enhance the separability between mangroves and water bodies, bare flats, and other coastal vegetation (Drusch et al., 2012*;* Hu et al., 2020*;* Zhang et al., 2023).

Sentinel-2 Level-2A surface reflectance imagery was obtained from the COPERNICUS/S2_SR_HARMONIZED collection in Google Earth Engine (GEE) (Gorelick et al., 2017). The dry-season window T1 covered 1 October-31 December 2024, and the wet-season window T2 covered 1 April-30 September 2024. All available acquisitions intersecting the region of interest within each window were included. Opaque cloud and cirrus pixels were masked by requiring QA60 bits 10 and 11 to equal zero, and reflectance values were divided by 10, 000. The masked collections were reduced directly with a pixel-wise median; no cloud-probability product, scene-level cloud threshold, monthly intermediate composite, missing-pixel filling, or tide-level filter was used. The ten selected bands were exported at 10 m in EPSG:4326 and subsequently aligned to the projected RGB grid during paired-sample construction.

The two Sentinel-2 windows were selected to contrast wet- and dry-season conditions within 2024, whereas the RGB imagery was acquired in October 2025. The data sources are therefore neither contemporaneous nor from the same year. The resulting features may contain useful seasonal contrast as well as variation caused by interannual change, residual atmosphere, water level, and temporary surface conditions. DCSF-Net uses these composites as contextual descriptors rather than as a recurrent or densely sampled time series; throughout this manuscript, spatiotemporal fusion refers specifically to the integration of RGB spatial features with two seasonal composites and their differences.

### 2.5 Reference-label production and quality control

Binary reference labels were manually delineated in October 2025 from the 0.3 m RGB imagery by three interpreters, and each delineation was reviewed by a second interpreter. Polygons interpreted as mangrove canopy formed the positive class; all remaining pixels were assigned to the background class after rasterization to the RGB grid. The task did not distinguish mangrove species or separate background subclasses. No independent field validation was conducted; accordingly, the manuscript uses the term reference labels rather than ground truth.

Label quality control combined manual visual review with an ancillary comparison against the 2020 mangrove extent layer of the Global Mangrove Watch *(1996-2020)* Version 3.0 dataset (Bunting et al., 2022). This product was used only to identify broad spatial inconsistencies and did not replace the 0.3 m manual interpretation or serve as field validation. Remaining uncertainty is expected to be greatest for sparse or young stands, mixed coastal vegetation, partially inundated margins, and gradual canopy transitions.

## 3 Methods

**Figure 2:**
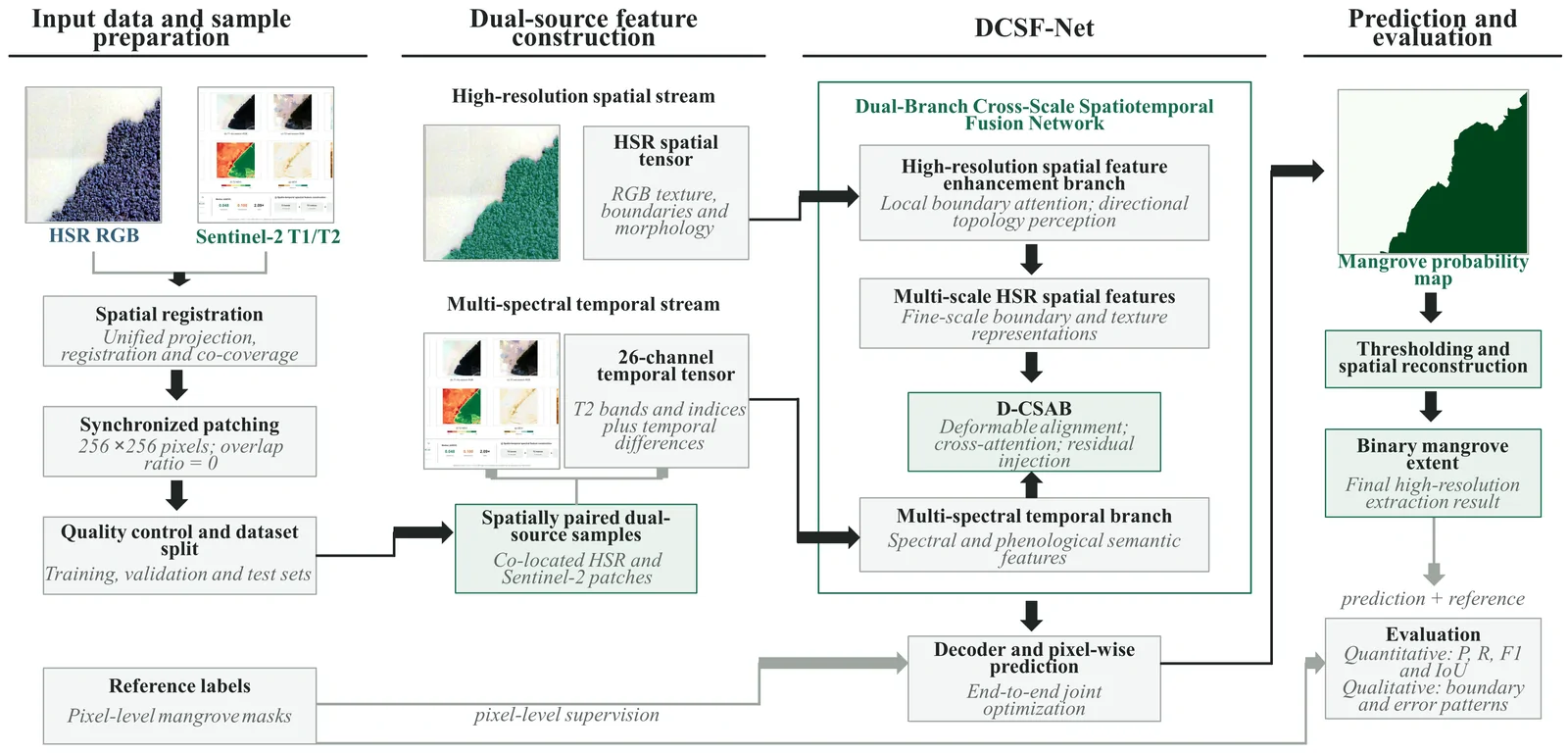
Workflow of this study. Black arrows indicate data flow, and gray arrows indicate supervision and validation using reference labels.

**Figure 2** summarizes the study workflow. Sentinel-2 bands, indices, and two-season differences were first calculated and aligned with the RGB coordinate grid. RGB imagery, the Sentinel-2 feature stack, and reference labels were then cropped using the same 76.8 m fishnet cells with a stride of 76.8 m and 0% overlap. DCSF-Net processed a 3 × 256 × 256 RGB tensor and a 26 × 256 × 256 Sentinel-2 tensor in separate branches. The RGB encoder-decoder generated the high-resolution spatial feature tensor, which was fused once with the upsampled Sentinel-2 feature through D-CSAB before prediction on the RGB grid. LBA and DTM were applied in parallel at the first three RGB encoder levels (Gorelick et al., 2017).

### 3.1 Extraction of multispectral and seasonal phenological features

Mangrove reflectance in intertidal zones varies with canopy condition, inundation, mudflat exposure, and water background. To supplement the limited spectral information in the RGB imagery, we used two Sentinel-2 seasonal composites from 2024: T1 (October-December, dry season) and T2 (April-September, wet season). We extracted ten multispectral bands, the normalized difference vegetation index (NDVI), enhanced vegetation index (EVI), and modified normalized difference water index (MNDWI), together with their seasonal differences. NDVI and EVI describe vegetation-related spectral variation, whereas MNDWI helps characterize water and wet-background differences (Rouse et al., 1974*;* Huete et al., 2002*;* Xu, 2006). The three spectral indices were calculated using **Equations 1**–**3**, as follows:

(1) $$NDVI=\frac{\rho_{NIR}-\rho_{Red}}{\rho_{NIR}+\rho_{Red}}$$

(2) $$EVI=2.5\times \frac{\rho_{NIR}-\rho_{Red}}{\rho_{NIR}+6\times \rho_{Red}-7.5\times \rho_{Blue}+1}$$

(3) $$MNDWI=\frac{\rho_{Green}-\rho_{SWIR}}{\rho_{Green}+\rho_{SWIR}}$$

where *ρ_Blue_*, *ρ_Green_*, *ρ_Red_*, *ρ_NIR_*, and *ρ_SWIR_* denote the reflectance of the corresponding Sentinel-2 bands.

**Figure 3:**
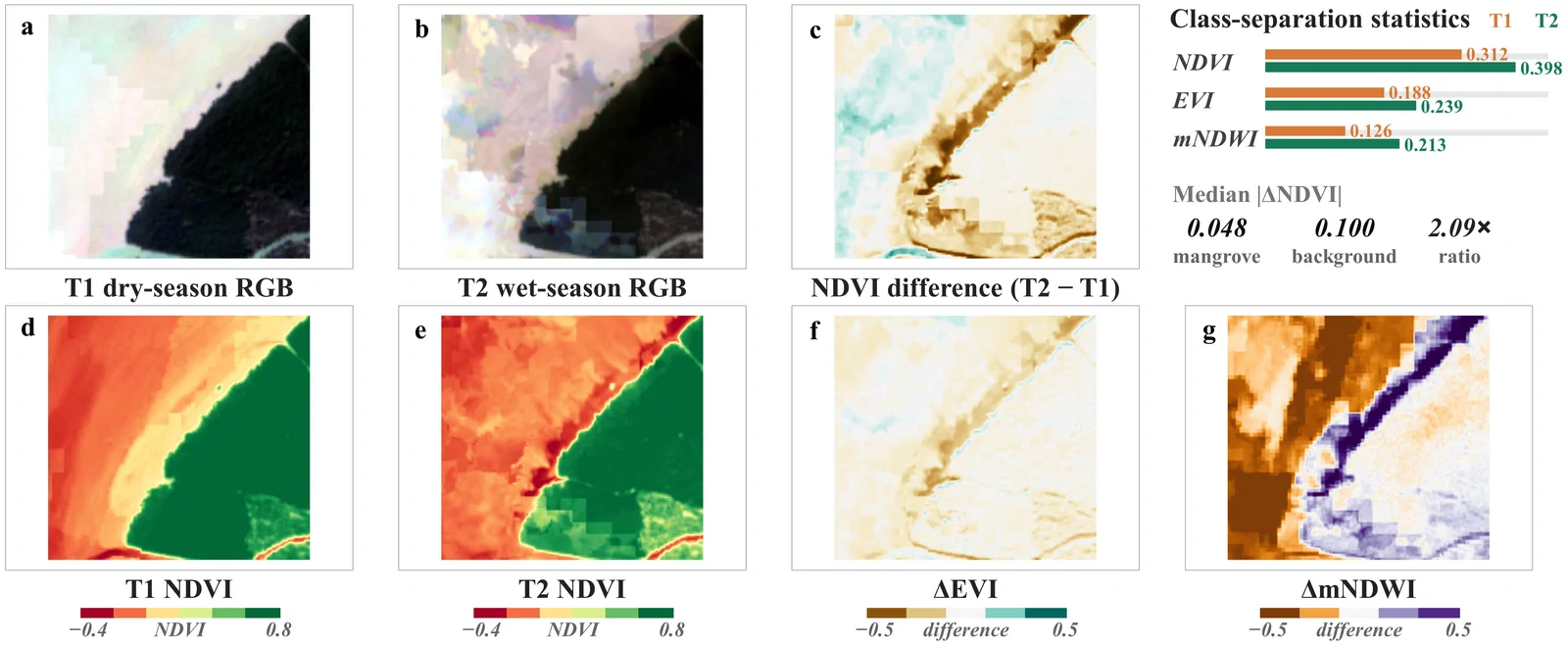
Mangrove phenological differences and inter-class separability derived from dual-temporal Sentinel-2 imagery. **(a, b)** True-color images from the T1 dry season and T2 wet season; **(c)** NDVI difference between the two dates; **(d, e)** spatial distributions of NDVI at T1 and T2; **(f, g)** temporal differences in EVI and MNDWI. The upper-right inset shows mangrove-background separability for NDVI, EVI, and MNDWI at the two dates, as well as the median temporal change in NDVI for mangrove and background pixels.

Most mangrove species are evergreen, and their seasonal spectral variation may differ from that of seasonal vegetation, exposed tidal flats, and water backgrounds. T1 represents dry-season conditions and T2 represents wet-season conditions; no direct physiological water-stress measurement was used. As shown in **Figure 3**, the two seasonal composites and their NDVI distributions differed. The absolute differences between median mangrove and background values for NDVI, EVI, and MNDWI were 0.312, 0.188, and 0.126 at T1 and 0.398, 0.239, and 0.213 at T2. The median seasonal NDVI change was 0.100 for background pixels and 0.048 for mangrove pixels. These observations motivated the use of band differences (ΔB) and index differences (ΔI) as complementary features (Tian et al., 2020*;* Hu et al., 2020). The band and vegetation-index differences were calculated using **Equations 4**, **5**, respectively, as follows:

(4) $$\Delta \rho =\rho_{T_{2}}-\rho_{T_{1}}$$

(5) $$\Delta VI=VI_{T_{2}}-VI_{T_{1}}$$

The Sentinel-2 input concatenated ten T2 bands, three T2 indices, ten T2-T1 band differences, and three T2-T1 index differences, yielding 26 channels. Retaining the wet-season reflectance together with wet-to-dry differences provides a seasonal state and a compact representation of interseasonal change without fitting a temporal sequence model. We use the term Sentinel-2 spectral-phenological feature tensor throughout the text, while spatiotemporal fusion refers to its integration with high-resolution RGB spatial features. The channel composition of the resulting 26-channel tensor is summarized in **Table 1**.

### 3.2 Dual-branch cross-scale fusion network

**Figure 4:**
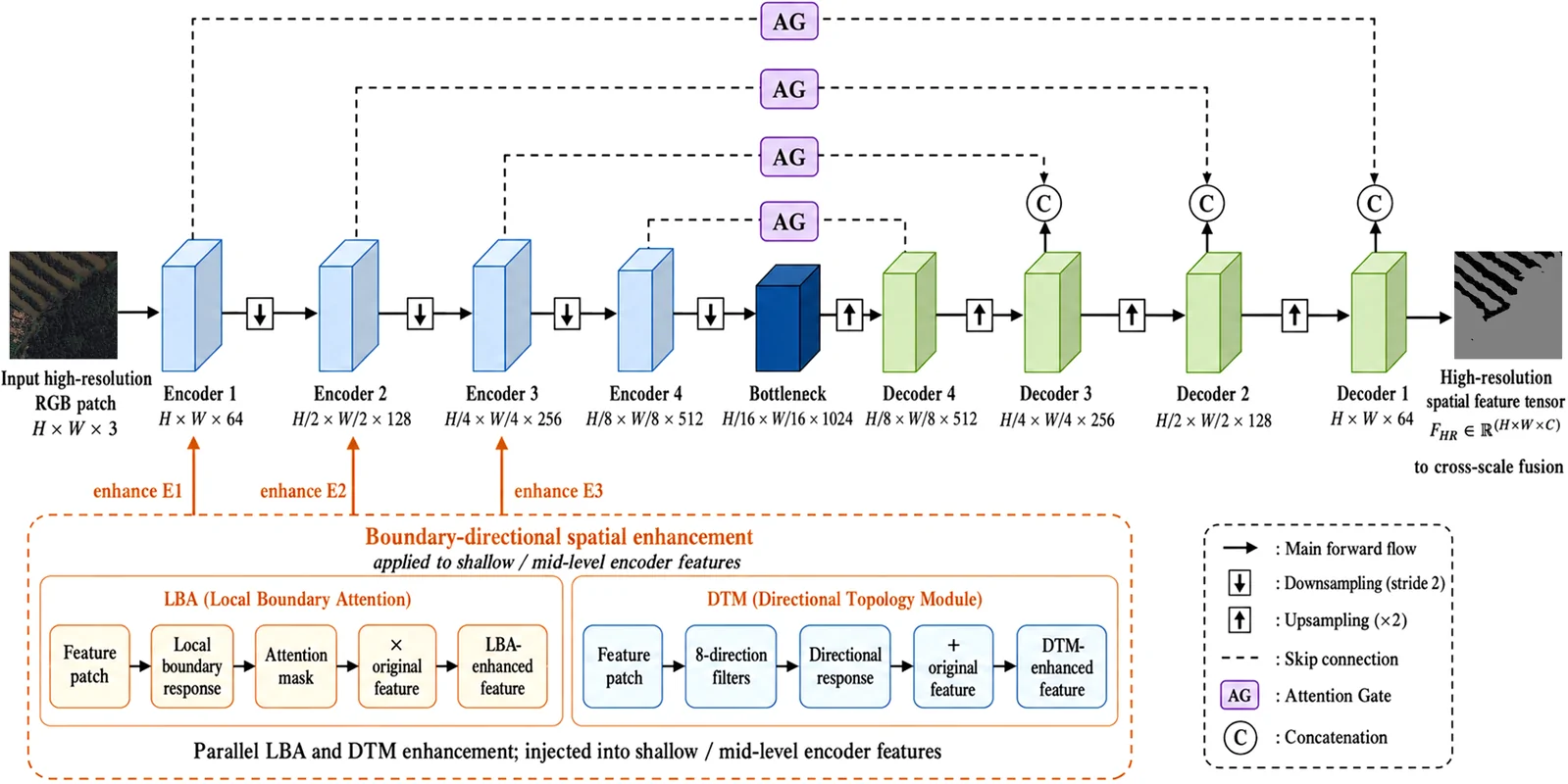
Schematic of the high-resolution spatial feature enhancement branch.

DCSF-Net contains a high-resolution RGB encoder-decoder, a Sentinel-2 spectral-phenological encoder, and one D-CSAB at the cross-scale fusion stage. As shown in **Figure 4**, the RGB encoder widths are 64, 128, 256, 512, and 1024 channels, followed by decoder widths of 512, 256, 128, and 64 channels. The Sentinel-2 encoder widths are 64, 128, 256, and 512 channels. After the RGB decoder produces the H × W × 64 high-resolution spatial feature tensor F_HR, D-CSAB receives F_HR together with the upsampled multispectral-temporal feature F_MS^up from the Sentinel-2 branch. The module aligns and fuses these representations before the prediction head. LBA and DTM are applied in parallel at the first three RGB encoder stages (64, 128, and 256 channels), and cross-source interaction occurs once after the RGB spatial branch.

The three modules serve distinct functions. LBA reweights local contrast responses in shallow and middle RGB features, DTM uses directional-response variance to emphasize anisotropic structures such as narrow or belt-like patches, and D-CSAB aligns and selects Sentinel-2 context after RGB decoding through offset prediction, deformable convolution, cross-attention, and residual injection. Their roles are complementary rather than interchangeable.

### 3.3 High-resolution spatial feature enhancement branch

The RGB branch follows a four-stage U-Net encoder-decoder. At the first three encoder stages (64, 128, and 256 channels), LBA uses a channel-reduction ratio of 4, while DTM applies eight grouped 3 × 3 directional filters, reduces the working channels to one quarter of the input width, computes the variance of directional responses, and maps that variance through a sigmoid gate. The modules are concentrated in shallow and middle stages to retain local boundary and directional information before strong spatial compression.

Specifically, to enhance the local difference representation between canopy edges and surrounding backgrounds, the network constructs neighborhood contrast responses on shallow and middle features. Let the feature at layer l be compressed along the channel dimension into a single-channel response map *S_l_*. The spatial attention weight *A_l_(p)* was calculated using Equation 6 from the local max–min contrast followed by lightweight convolutional mapping, as follows:

(6) $$A_{l}(p)=\text{Sigmoid}(\text{Conv}({\text{max}}_{q\in \Omega_{k}(p)}S_{l}(q)-{\text{min}}_{q\in \Omega_{k}(p)}S_{l}(q)))$$

The original features are multiplied element-wise by the LBA weights to strengthen local responses near annotated canopy edges, tidal channels, and land-water transitions.

DTM characterizes anisotropy by comparing responses from eight discrete directional filters. Let the filter responses at pixel p be denoted by the terms defined below. The directional-response variance was calculated using **Equation 7**, as follows:

(7) $$D_{l}\left(p\right)=\frac{1}{N}\sum_{i=1}^{N}{\left[R_{i}\left(p\right)-\frac{1}{N}\sum_{j=1}^{N}R_{j}\left(p\right)\right]}^{2}$$

Large differences among directional responses produce a higher DTM weight, whereas more isotropic responses produce a lower weight. The resulting gate is applied to the LBA features at the first three encoder levels to retain directional evidence for narrow and belt-like structures.

In addition, during the decoding stage, an attention-gating mechanism is deployed at skip nodes at each level to prevent non-vegetated intertidal background noise from being transmitted to high-level features through skip connections. This mechanism uses the deep high-level semantic tensor as a gating guidance signal and generates two-dimensional spatial gating weights through independent linear projection and pixel-wise alignment with shallow spatial features.

### 3.4 Deformable cross-scale attention bridge

D-CSAB is used once after the RGB encoder-decoder produces the high-resolution spatial feature tensor F_HR. It couples F_HR with the upsampled Sentinel-2 multispectral-temporal feature F_MS^up while retaining the RGB grid as the output geometry. Its two computational stages are offset-guided deformable alignment and cross-attention with zero-initialized residual injection.

#### 3.4.1 Deformable pixel alignment based on offset-field prediction

**Figure 5:**
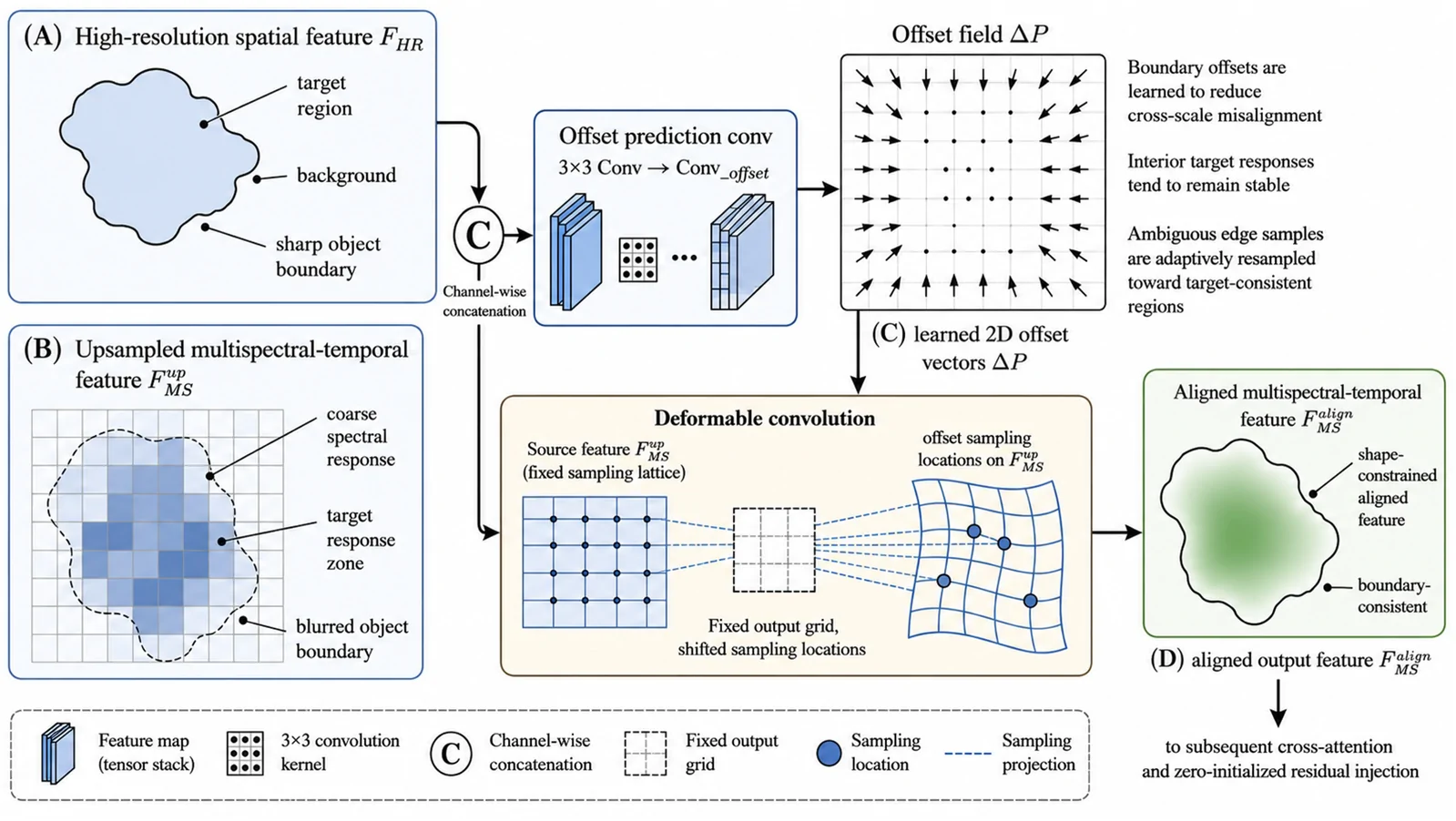
Schematic of nonlinear deformable alignment in D-CSAB. **(A)** High-resolution spatial feature with sharp target boundaries. **(B)** Upsampled multispectral-temporal feature with coarse spectral responses and blurred boundaries. **(C)** Learned two-dimensional offset vectors used to reduce cross-scale misalignment. **(D)** Aligned multispectral-temporal output feature with improved shape and boundary consistency.

As illustrated in **Figure 5**, the Sentinel-2 multispectral-temporal feature is first upsampled to the spatial dimensions of F_HR. F_HR and F_MS^up are concatenated channel-wise and passed through a 3 × 3 offset-prediction convolution to estimate a two-dimensional offset field ΔP. Deformable convolution then samples F_MS^up at the learned continuous offset locations by bilinear interpolation, producing the aligned feature F_MS^align. F_HR forms the query, while F_MS^align forms the key and value. A learnable scalar initialized to zero controls residual injection of the attended Sentinel-2 representation into the high-resolution RGB feature.

Because the offsets are predicted jointly from the high-resolution target geometry and the coarser Sentinel-2 response, the learned sampling locations are intended to reduce cross-scale misalignment and produce an aligned feature that is boundary-consistent with the target regions shown in **Figure 5**. This operation acts in the learned feature space and does not create native 0.3 m multispectral measurements.

#### 3.4.2 Cross-attention mechanism and zero-initialized residual injection

After deformable convolution, D-CSAB uses cross-attention to control which aligned Sentinel-2 features are injected into the high-resolution RGB feature. A 1 × 1 convolution maps F_HR to the query Q, and separate 1 × 1 convolutions map F_MS^align to the key K and value V. The query, key, and value tensors were calculated using **Equation 8**, as follows:

(8) $$Q=W_{q}\left(F_{\text{HR}}\right),\;K=W_{k}\left({\tilde{F}}_{\text{MS}}\right),\;V=W_{v}\left({\tilde{F}}_{\text{MS}}\right)$$

After the above tensors are reshaped and transposed, spatial-spectral affinity weights are calculated by matrix multiplication and then applied to the value tensor V to output the spatially modulated multispectral interaction feature *F_attn_* was calculated using **Equation 9**, as follows:

(9) $$F_{\text{attn}}=Softmax\left(\frac{QK^{T}}{\sqrt{d_{k}}}\right)V$$

where the scaling term is determined by the feature-channel dimension. This stage uses high-resolution spatial evidence from F_HR to modulate the contribution of Sentinel-2 context while preserving the RGB grid as the source of output geometry.

A zero-initialized residual injection (ZRI) weight controls the contribution of the fused Sentinel feature. The scalar is initialized to zero so that optimization begins from the RGB pathway and learns the Sentinel contribution gradually. The fused feature was calculated using **Equation 10**, as follows:

(10) $$F_{\text{fusion}}=F_{\text{HR}}+\alpha \cdot F_{\text{attn}}$$

When the residual weight is zero, the attended Sentinel-2 feature does not contribute to the fused high-resolution feature, so optimization begins from F_HR. As optimization proceeds, the learnable scalar is updated jointly with the network and controls the magnitude of Sentinel-2 feature injection. This design provides a stable optimization path without imposing a predetermined fusion ratio.

### 3.5 Accuracy assessment

To evaluate the performance of the mangrove mapping methods, this study used four widely adopted quantitative metrics: precision (P), recall (R), F1-score (F1), and intersection over union (IoU). Precision represents the percentage of correctly predicted mangrove regions among all regions predicted as mangroves. Recall represents the proportion of correctly predicted mangrove results relative to the total reference mangrove area. The F1-score is the harmonic mean of precision and recall. IoU represents the ratio of the intersection to the union between the predicted mangrove region and the reference region. These metrics are widely used in semantic segmentation and remote-sensing classification e*valuation* (Powers, 2011*;* Everingham et al., 2010). Based on true positive (TP), false positive (FP), false negative (FN), and true negative (TN), the four metrics were calculated using **Equations 11**–**14**, as follows:

(11) $$Precision=\frac{TP}{TP+FP}$$

(12) $$Recall=\frac{TP}{TP+FN}$$

(13) $$F1=2\times \frac{Precision\times Recall}{Precision+Recall}$$

(14) $$IoU=\frac{TP}{TP+FP+FN}$$

## 4 Experiments and results

### 4.1 Experimental setup

#### 4.1.1 Dataset preparation and partitioning

A total of 7, 610 unique RGB-Sentinel-reference-label pairs were generated using the non-overlapping fishnet. Partitioning was performed after cropping and manual label-quality review. The pairs were shuffled once with split seed 42 and assigned at an 80:10:10 ratio to training (6, 088), validation (761), and test (761) subsets. The assignment lists were fixed for all reported experiments. Each stored RGB patch, label patch, and reference-label pixel occurs in only one subset; because the fishnet stride equals the patch width, no stored high-resolution windows overlap across subsets.

This design ensures sample-level exclusivity but is not equivalent to geographic-block validation. Because assignment was random after cropping, immediately adjacent fishnet cells may occur in different subsets, and multiple cells may share the support of a native 10–20 m Sentinel-2 pixel after resampling. **Figure 1** shows the geographic coverage of the sampled mangrove areas; a separate subset-location map was not added because the random patch assignment would intermix the three subsets within the same sampled regions. The reported evaluation should therefore be interpreted as a random non-overlapping patch test rather than a geographically independent transfer test.

**Table 1:** Channel composition of the Sentinel-2 spectral-phenological feature tensor.

Feature category	Feature variables	Channels
T2 Raw Bands	B2, B3, B4, B5, B6, B7, B8, B8A, B11, B12	10
T2 Spectral Indices	NDVI, MNDWI, EVI	3
Δ Bands: T2−T1	ΔB2, ΔB3, ΔB4, ΔB5, ΔB6, ΔB7, ΔB8, ΔB8A, ΔB11, ΔB12	10
Δ Indices: T2−T1	ΔNDVI, ΔMNDWI, ΔEVI	3
Total		26

#### 4.1.2 Experimental environment and hyperparameter configuration

All experiments used one NVIDIA GeForce RTX 4090 GPU (24 GB), Windows 11, Python 3.10, and PyTorch 2.1. Models were trained for 200 epochs with batch size 8, automatic mixed precision, AdamW, an initial learning rate of 1 × 10−4, weight decay of 1 × 10−4, and cosine learning-rate decay. The objective combined 0.5 binary cross-entropy and 0.5 Tversky loss, with Tversky α = 0.7, β = 0.3, and smoothing = 1 × 10−6. Predictions were thresholded at 0.5. Training augmentation comprised random scaling from 0.8 to 1.2 (p = 0.5), a 256 × 256 random crop, horizontal flipping (p = 0.5), rotation within ±30° (p = 0.5), RGB color jitter (brightness, contrast, and saturation = 0.2; hue = 0.1; p = 0.5), and Gaussian blur (5 × 5 kernel, σ = 0.1-2.0; p = 0.3). Geometric transforms were synchronized between imagery and labels.

The dataset split used seed 42. The archived training configuration did not set a separate global seed for all stochastic training operations, so exact run-to-run determinism is not claimed (Loshchilov and Hutter, 2019; Loshchilov and Hutter, 2017). The best checkpoint was selected according to the highest IoU on the validation set, and the test set was used for final evaluation (Salehi et al., 2017; Lin et al., 2017).

### 4.2 Comparative experimental results

The experiments address two complementary questions. **Table 2** compares the complete RGB-Sentinel-2 DCSF-Net system with evaluated RGB-only segmentation systems under the same data split; this system-level comparison does not isolate input information from architecture. **Table 4** provides the matched-input controls: direct fusion versus D-CSAB, removal of ZRI, and joint removal of LBA and DTM all retain both input sources. The variant without the Sentinel-2 branch changes both the available information and the fusion pathway and is therefore interpreted as a combined system ablation.

**Table 2:** Quantitative comparison with evaluated RGB-only semantic segmentation baselines on the test set.

Type	Method	P (%)	R (%)	F1 (%)	IoU (%)
Encoder-decoder	U-Net	89.47	90.28	89.87	81.61
	UNet++	90.54	89.41	89.97	81.77
Multi-scale/context	PSPNet	90.12	89.65	89.88	81.63
	DeepLabV3+	91.24	91.03	91.13	83.71
	HRNet	92.41	91.56	91.98	85.15
	DANet	92.87	90.74	91.79	84.83
	OCRNet	92.15	91.10	91.62	84.53
Transformer-based	UNetFormer	92.04	92.45	92.24	85.60
	SegFormer	92.56	93.24	92.90	86.74
**Proposed**	**DCSF-Net**	**95.75**	**95.61**	**95.68**	**91.72**

Bold values indicate the best performance in each column.

**Table 4:** Ablation experiments for key modules.

Model variant	S2 branch	D-CSAB	ZRI	LBA + DTM	P (%)	R (%)	F1 (%)	IoU (%)
**DCSF-Net**	**√**	**√**	**√**	**√**	**95.75**	**95.61**	**95.68**	**91.72**
Removing the Sentinel-2 spectral-phenological branch	×	×	×	✓	93.70	93.25	93.47	87.75
Replacing D-CSAB with direct fusion	✓	×	✓	✓	94.20	93.68	93.94	88.52
Removing ZRI	✓	✓	×	✓	94.88	94.44	94.66	89.86
Removing LBA and DTM	✓	✓	✓	×	92.94	92.42	92.68	86.36

S2 branch denotes the Sentinel-2 spectral-phenological branch, D-CSAB denotes the Deformable Cross-Scale Attention Bridge, ZRI denotes zero-initialized residual injection, and LBA+DTM denotes the combined local boundary attention and directional topology module. Bold values indicate the best performance in each column.

**Table 3:** Performance of representative models on the validation set.

Model	P (%)	R (%)	F1 (%)	IoU (%)
DANet	89.00	87.30	88.20	78.83
HRNet	89.70	88.30	89.00	80.15
UNetFormer	90.20	89.50	89.90	81.60
SegFormer	91.30	90.70	91.00	83.54
**DCSF-Net**	**96.65**	**91.27**	**93.88**	**88.47**

Bold values indicate the best performance in each column.

#### 4.2.1 Quantitative analysis of models

DCSF-Net achieved an F1 of 95.68% and an IoU of 91.72% on the test set. Among the evaluated RGB-only baselines, HRNet achieved an F1 of 91.98% and an IoU of 85.15%, while SegFormer achieved an F1 of 92.90% and an IoU of 86.74%. These comparisons demonstrate the performance of the complete dual-source system; the matched-input ablations are used to assess the contribution of the tested fusion design.

The complete DCSF-Net system achieved higher scores than the evaluated RGB encoder-decoder models. U-Net and UNet++ achieved IoU values of 81.61% and 81.77% and F1 values of 89.87% and 89.97%, respectively. These results show a system-level advantage under the present inputs and split, without attributing the entire difference to any single module.

Among the evaluated multi-scale and context-enhanced RGB baselines, HRNet achieved the highest F1 (91.98%) and IoU (85.15%). The performance difference is consistent with the complementary value of RGB spatial detail and Sentinel-2 spectral-seasonal context, while the matched-input ablations in **Table 3** provide the more direct test of the fusion architecture.

**Figure 6:**
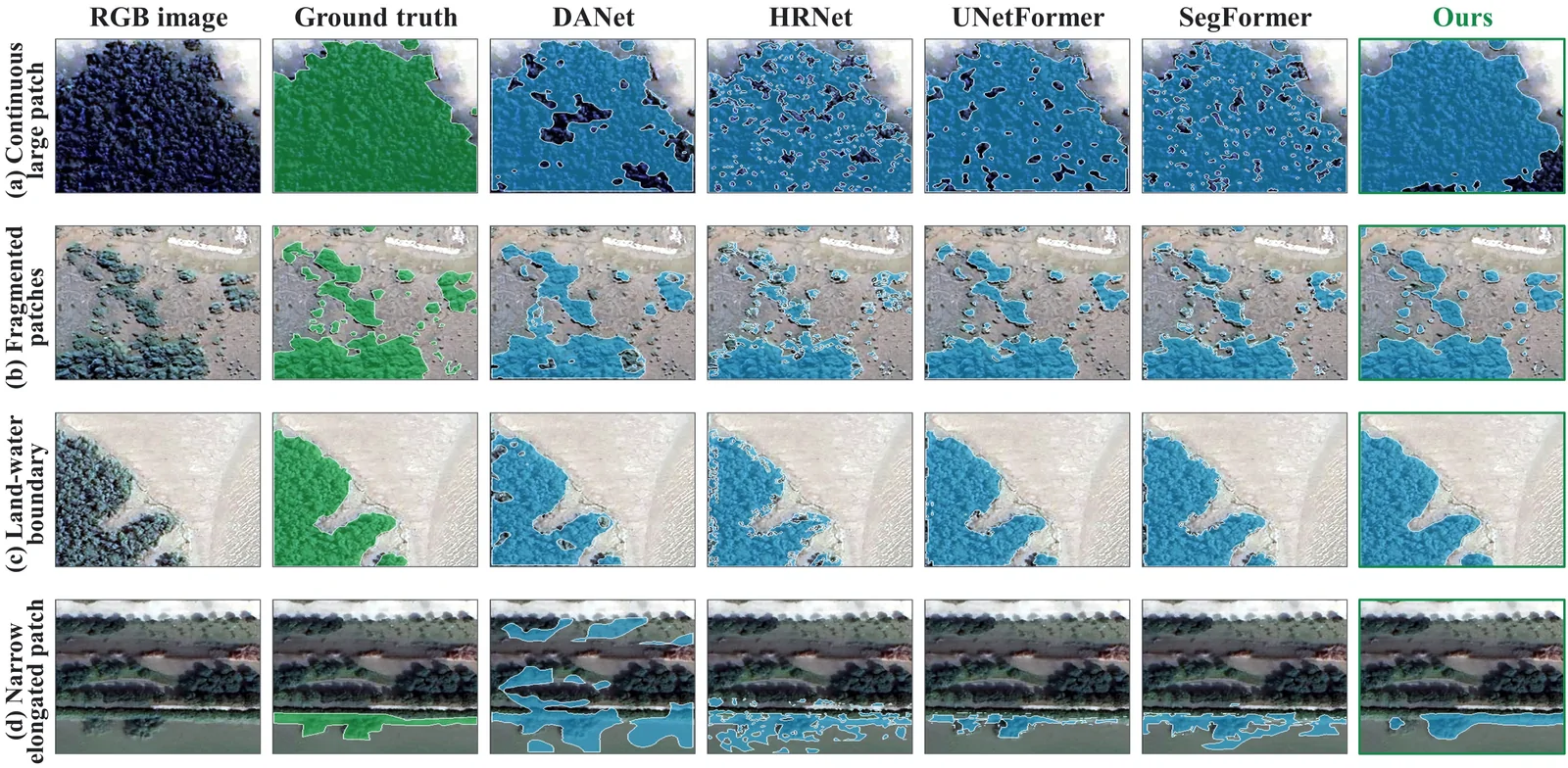
Qualitative comparison of genuine model predictions in four purposively selected held-out cases. Blue translucent regions indicate predicted mangrove extents, and green regions indicate reference labels. The examples illustrate error modes and are not a statistically representative sample.

**Figure 7:**
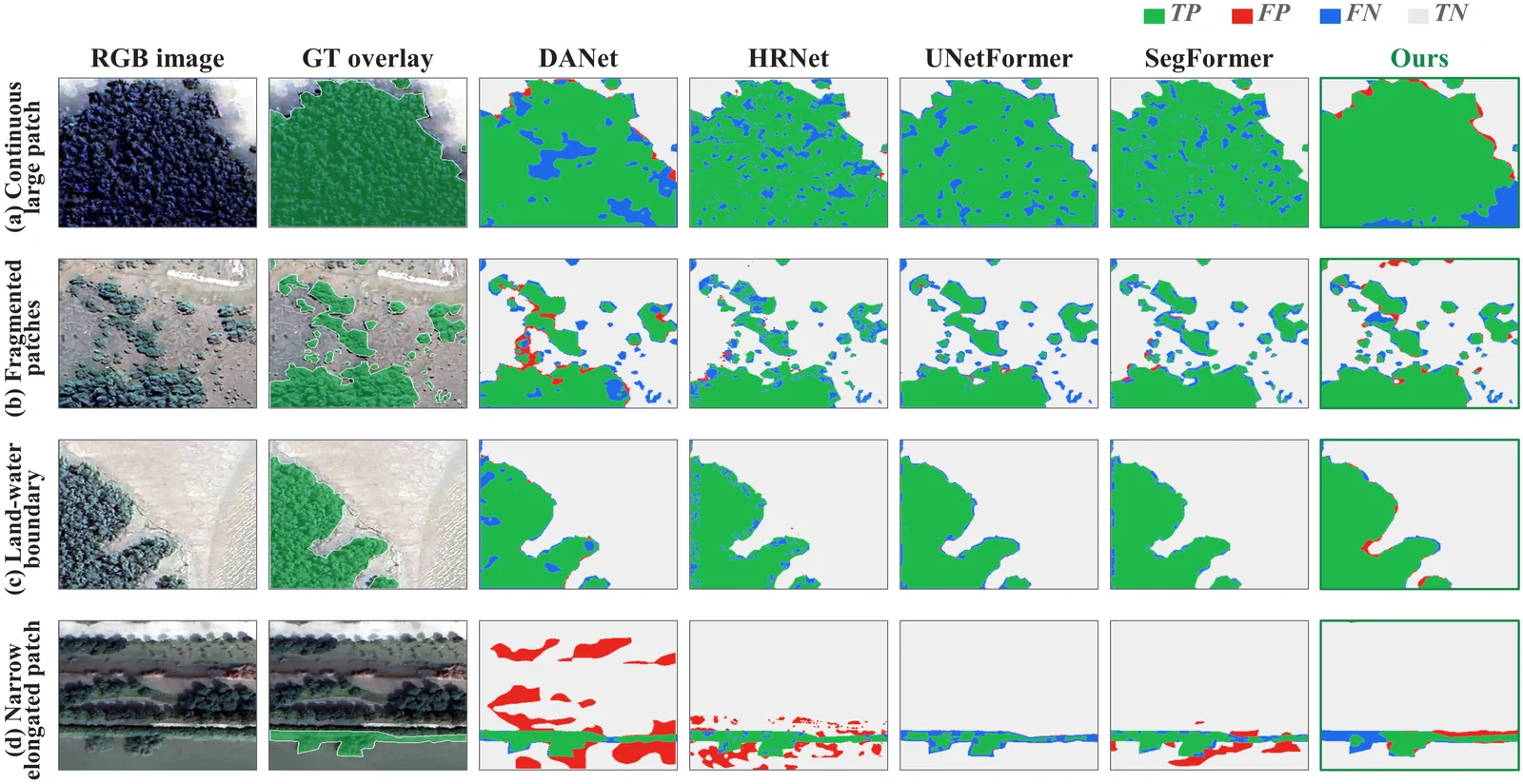
Error decomposition for the same genuine held-out predictions. Green, red, blue, and light gray indicate true positive (TP), false positive (FP), false negative (FN), and true negative (TN) pixels relative to the reference labels.

SegFormer was the strongest evaluated Transformer-based RGB baseline, with an F1 of 92.90% and an IoU of 86.74%. DCSF-Net exceeded these values by 2.78 and 4.98 percentage points, respectively. The larger IoU difference indicates improved regional overlap, while boundary-specific effects are examined qualitatively in **Figures 6** and **7** because no boundary metric was added.

**Figure 8:**
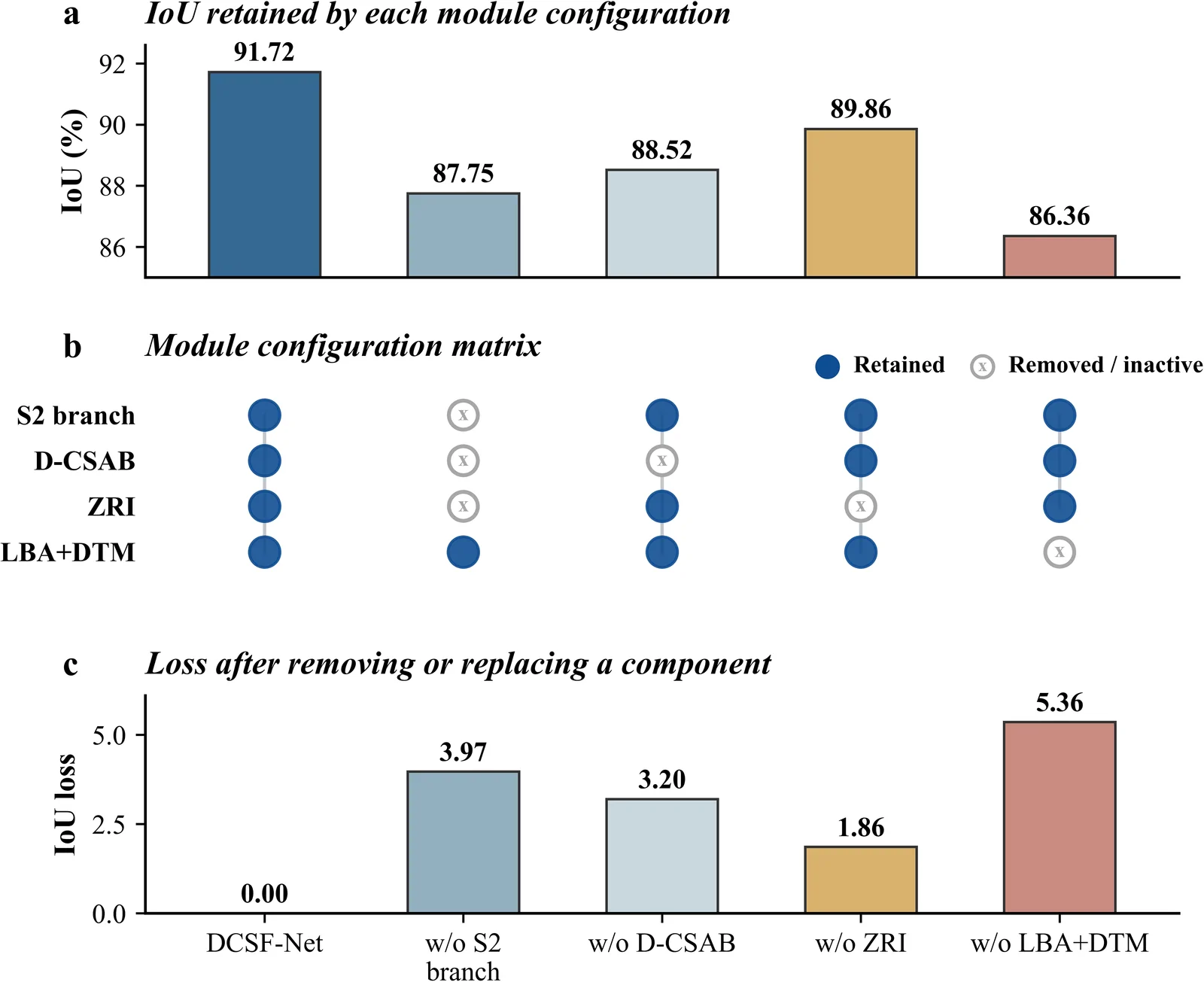
Ablation performance comparison of key modules. **(a)** IoU retained by each module configuration. **(b)** Module configuration matrix showing retained, removed, or inactive components; filled circles denote retained modules, whereas open circles denote removed or inactive modules. In the w/o S2 branch variant, removal of the Sentinel-2 spectral-phenological branch makes D-CSAB and ZRI inactive because both operate on the dual-branch fusion pathway. **(c)** IoU decrease of each variant relative to DCSF-Net. w/o denotes without; S2 branch denotes the Sentinel-2 spectral-phenological branch.

As shown in **Table 4**, DCSF-Net achieved validation-set precision, recall, F1, and IoU values of 96.65%, 91.27%, 93.88%, and 88.47%, respectively, compared with the four comparison models that performed best on the test set. The validation and test subsets were drawn by the same random procedure but were not stratified by geographic block, mangrove-area proportion, or scene difficulty. Their score difference is therefore most plausibly interpreted as finite-sample variation under random allocation rather than as evidence of a verified geographic or categorical mechanism.

#### 4.2.2 Qualitative analysis of models

**Figures 6** and **7** use genuine predictions generated by the final trained checkpoints on held-out samples. The four examples were selected purposively from the available predictions using reference-label morphology to cover (i) a relatively continuous stand, (ii) fragmented small patches, (iii) a land-water transition, and (iv) a narrow belt. This selection was designed to expose different spatial error modes rather than estimate their prevalence. The examples are therefore illustrative and are not treated as a statistically representative sample of the full evaluation subset.

**Figure 6** overlays the predicted mangrove extents from the evaluated models and the reference labels. The continuous-stand case illustrates internal omissions, the fragmented case illustrates missed small patches and local adhesion, the land-water case illustrates feature alignment near a transition, and the narrow-belt case illustrates continuity along elongated targets.

In the continuous-stand example, the evaluated models differed in internal omission and edge consistency. DCSF-Net produced a more continuous region and fewer visible internal gaps than the displayed RGB-only baselines. This is a qualitative comparison for the selected case and should not be interpreted as a boundary-accuracy statistic.

In the fragmented and narrow-belt examples, DCSF-Net visually retained more small or elongated labeled regions than the displayed baselines. Residual errors remained around low-contrast transitions. These observations are consistent with, but do not independently prove, the roles assigned to the RGB morphology modules and the Sentinel-2 fusion pathway.

In the continuous-stand example, visible differences mainly involved internal omissions and edge consistency. In the fragmented and narrow-belt examples, the principal visible failures were missed small components, local adhesion, and discontinuity along elongated targets. At the land-water interface, false-positive and false-negative pixels were concentrated around low-contrast transitions. Across the four examples, exposed flats, water edges, shadows, aquaculture-pond edges, and non-mangrove coastal vegetation were plausible confusion contexts on visual inspection. Because the binary reference labels do not encode these background subclasses, this is an image-based error interpretation rather than a category-specific confusion analysis.

DCSF-Net showed more localized visible errors than the displayed baselines in these selected cases, but omissions and offsets remained in narrow regions and ecological transition zones. We therefore describe the apparent improvement in fragmentation and boundary continuity as qualitative. No claim of dataset-wide boundary superiority is made, and Boundary F1, Boundary IoU, or a buffer-based metric remains necessary to test that hypothesis quantitatively.

### 4.3 Ablation experimental results

**Figure 8** summarizes the retained IoU, module configurations, and IoU decreases for the ablation variants. All four ablation variants in **Table 4** were genuinely retrained and evaluated using the same 6, 088/761/761 partition and the same optimization settings as the complete model. The direct-fusion, without-ZRI, and without-LBA+DTM variants retained both RGB and Sentinel-2 inputs. They therefore control the input information while changing the tested fusion or RGB-branch component. The without-Sentinel-2 variant removes both the additional data source and the modules that depend on it; its performance difference is not interpreted as a pure architectural effect.

The complete model achieved P, R, F1, and IoU values of 95.75%, 95.61%, 95.68%, and 91.72%, respectively. Removing the Sentinel-2 branch reduced IoU to 87.75% and F1 to 93.47%. Because this ablation removes both the additional data source and the associated fusion path, the difference quantifies their combined contribution and is not attributed solely to architecture.

Relative to the complete model IoU of 91.72%, direct fusion reduced IoU to 88.52% (−3.20 percentage points), removing ZRI reduced it to 89.86% (−1.86 points), and removing LBA+DTM reduced it to 86.36% (−5.36 points). These matched-input comparisons support the tested D-CSAB configuration, gradual residual injection, and the combined RGB morphology enhancement, respectively. They do not rank D-CSAB against every possible multi-source fusion design, and the combined LBA+DTM ablation cannot separate the individual effects of LBA and DTM. (Ronneberger et al., 2015; Zhou et al., 2018; Zhao et al., 2017; Chen et al., 2018; Sun et al., 2019; Fu et al., 2019; Yuan et al., 2020b; Wang et al., 2022; Xie et al., 2021). Removing Sentinel-2 reduced IoU to 87.75% and quantifies only the combined value of the additional source and its fusion path.

Replacing D-CSAB with direct fusion retained the same RGB and Sentinel-2 inputs and reduced IoU from 91.72% to 88.52%. This matched-input comparison supports the contribution of content-adaptive resampling, cross-attention, and residual fusion relative to the tested direct-fusion configuration. It does not establish superiority over all possible multi-source fusion architectures. Related mangrove segmentation studies have also evaluated DeepLabV3+, UPerNet, Swin Transformer, and SegFormer (Wang et al., 2023; Wang et al., 2024b).

Removing ZRI while retaining both inputs yielded an IoU of 89.86% and an F1 of 94.66%, 1.86 and 1.02 percentage points below the complete model. This matched-input ablation is consistent with a beneficial optimization role for gradual residual injection.

Removing LBA and DTM while retaining the dual-source input produced an IoU of 86.36%, 5.36 percentage points below the complete model. This result supports the joint contribution of the tested RGB-branch morphology modules within DCSF-Net, although the combined ablation does not separate LBA from DTM individually.

## 5 Discussion

### 5.1 Scale effects and boundary definition in fine-scale mangrove mapping

Fine-scale mangrove mapping is fundamentally affected by observation scale. Mangroves occupy transition zones among tidal channels, mudflats, water bodies, aquaculture areas, and terrestrial vegetation, and their ecological edges do not always appear as stable geometric lines. At 10–30 m resolution, mixed pixels can combine canopy, water, exposed sediment, and other vegetation, shifting apparent boundaries and reducing the detectability of narrow belts and small fragments (Kuenzer et al., 2011*;* Zhang et al., 2025). Tidal state further changes the visible land-water interface and can alter the spectral background surrounding the canopy (Rogers et al., 2017).

The 0.3 m RGB imagery alleviates part of this scale effect by preserving canopy texture, patch morphology, tidal-channel edges, and shoreline context. Nevertheless, young stands, sparse crowns, partially inundated margins, and gradual vegetation transitions remain ambiguous for both interpretation and prediction. The reported IoU should therefore be understood as agreement with the reference labels at the adopted 0.3 m mapping scale, rather than as direct measurement of a unique ecological boundary.

Overall P, R, F1, and IoU summarize regional classification performance but do not isolate boundary behavior. Boundary IoU, Boundary F1, buffer-based accuracy, and patch-size-stratified recall would provide a more direct assessment of continuous stands, small fragments, narrow belts, and tidal-channel margins in future work (Cheng et al., 2021).

### 5.2 Seasonal context, tidal variability, and mixed-pixel uncertainty

The two data sources contribute different forms of evidence. RGB imagery supplies fine spatial geometry but offers only visible-band information, making mangroves difficult to distinguish from shadows, turbid water, exposed flats, aquaculture-pond edges, and other coastal vegetation. Sentinel-2 contributes red-edge, near-infrared, shortwave-infrared, water-related, and seasonal information that can stabilize semantic discrimination in these backgrounds (Hu et al., 2020*;* Tian et al., 2020). The ablation results are consistent with this complementarity: removing the Sentinel-2 branch reduced IoU, and replacing D-CSAB with direct fusion reduced performance while retaining both inputs.

These benefits are accompanied by temporal and spatial uncertainty. The 2024 seasonal composites precede the October 2025 RGB imagery, and no tide-level filter was applied. Their differences may therefore reflect seasonal phenology together with interannual change, residual atmospheric effects, water-level variation, and temporary surface conditions. In addition, resampling native 20 m bands to the 10 m export grid and subsequently to the network interface cannot remove mixed-pixel limitations. The Sentinel-2 pathway should consequently be interpreted as providing contextual spectral-seasonal evidence rather than sub-meter spectral detail (Simarmata et al., 2024).

### 5.3 From segmentation accuracy to mapping reliability and application

The strong test-set scores and the qualitative examples indicate that DCSF-Net can support fine-scale delineation of continuous stands, fragmented patches, and narrow coastal belts within the sampled Greater Bay Area. Such maps are relevant to inventory updating, restoration-site assessment, shoreline management, and detection of small or discontinuous mangrove patches that are poorly represented in medium-resolution products. However, the RGB-only baseline comparison evaluates the complete system, while structural attribution rests on the matched-input ablations. Moreover, the random patch split allows adjacent cells to enter different subsets and does not test transfer to geographically separated coasts. Operational use therefore requires geographically blocked validation and independent assessment of false positives in complex non-mangrove backgrounds.

### 5.4 Limitations and future work

Several limitations remain. The manually interpreted binary labels were reviewed by multiple interpreters but were not validated with independent field data and do not distinguish species or background subclasses. The 2024 Sentinel-2 composites and October 2025 RGB imagery are temporally mismatched, no tide filter was used, and native 10–20 m mixed pixels constrain cross-scale correspondence. The random non-overlapping patch split prevents duplicate RGB pixels across subsets but does not establish geographic transferability. Future validation should therefore include geographically blocked and cross-region tests, field-supported or independently reinterpreted labels, tide-aware or acquisition-matched compositing, and boundary- and patch-scale metrics. Salt-marsh invasion can interact with mangrove fragmentation and alter boundary interpretation (Zhang et al., 2021), while fragmentation can also affect canopy structure, leaf area index, and gross primary productivity (Kanniah et al., 2021). These ecological changes may further complicate reference-label interpretation and cross-region model transfer.

Model complexity is a second practical limitation. DCSF-Net combines a high-resolution encoder-decoder, a Sentinel-2 branch, deformable feature alignment, cross-attention, and residual injection, which may increase memory use and inference time relative to lightweight segmentation models. Future work should quantify computational cost and tiled-prediction stability and explore lightweight backbones, knowledge distillation (Hinton et al., 2015), sparse attention, model pruning (Han et al., 2016), and multi-scale inference for large-area monitoring. Future work could also incorporate predictive uncertainty estimation (Gal and Ghahramani, 2016; Kendall and Gal, 2017).

## 6 Conclusions

This study developed DCSF-Net to integrate 0.3 m RGB imagery with a 26-channel Sentinel-2 spectral-phenological feature tensor for fine-scale mangrove mapping. The RGB pathway preserves local boundary contrast and directional morphology, while D-CSAB fuses the high-resolution spatial feature tensor output by the RGB encoder-decoder with the upsampled Sentinel-2 feature through offset-guided deformable convolution, cross-attention, and zero-initialized residual injection. DCSF-Net achieved an F1 of 95.68% and an IoU of 91.72% on the test set and outperformed the evaluated RGB-only baselines. Matched-input ablations supported the implemented D-CSAB relative to direct fusion, the optimization role of ZRI, and the joint contribution of LBA and DTM. The results show that complementary spectral-seasonal context can improve high-resolution mangrove mapping within the present study area and experimental design, while geographically blocked testing, temporally matched data, field-supported labels, and boundary-specific metrics remain priorities for further validation.

## Data Availability

The data and source code supporting the findings of this study are available from the corresponding authors upon reasonable request for academic research and scholarly exchange.
